# Reducing electric energy consumption in linear Fresnel collector solar fields coupled to thermal desalination plants by optimal mirror defocusing

**DOI:** 10.1016/j.heliyon.2018.e00813

**Published:** 2018-09-28

**Authors:** Mohamed Alhaj, Sami G. Al-Ghamdi

**Affiliations:** Division of Sustainable Development, College of Science and Engineering, Hamad Bin Khalifa University, Qatar Foundation, Doha, Qatar

**Keywords:** Energy, Mechanical engineering

## Abstract

In many parts of the world, desalination is the only viable and economic solution to the problem of fresh water shortage. The current commercial desalination technologies rely on fossil fuels and are thus associated with high greenhouse gas emissions that are a major cause of climatic changes. Solar thermal-driven multi-effect distillation with thermal vapor compression is a clean alternative to conventional desalination technologies. To comprehend this process, as well as its features and limitations, extensive modeling is required. In this work, we proposed a plant design based on a solar field with a linear Fresnel collector that supplies heat to a multi-effect distillation plant with thermal vapor compression. The solar desalination plant model is implemented in the Engineering Equation Solver (EES). The system performance is investigated and a control strategy for reducing electric pumping is proposed. Results showed that 1 m^2^ of the solar field produces 8.5 m^3^ of distillate per year. The proposed control strategy resulted in a 40% reduction in electric pumping energy. Our results highlight the versatility of the linear Fresnel collector when coupled with thermal desalination.

## 1 Introduction

Providing drinking water is one of the greatest challenges of our times. In many parts of the world, desalination is the only viable and economic solution to the problem of fresh water shortage. This is particularly applicable to the Gulf Cooperation Council (GCC) region, which has the largest installed capacity of desalination plants in the world, amounting to 38% of the global capacity [1, 2] Most desalination plants in the GCC region operate using multistage flash (MSF) and multi-effect distillation (MED) with thermal vapor compression (TVC). The reverse osmosis process (RO), however, is gaining increased popularity and currently has 29% of the market share in the GCC region [3]. By 2013, 3,732 online plants were operating in the GCC region that produced 29,503 Mm^3^ of fresh water per day [4]. Water desalination is an energy intensive process, and hence requires large amounts of fuel. It was estimated that in 2012, desalination plants in the GCC region consumed 3.49 mega Joules of fuel per day [5]. Furthermore, in 2014, the GCC countries spent $15.9 billion in fuel costs for desalination [6]. At a global scale, owing to the population increase and increased demand for food, the demand for fresh water will continue to rise. This will impose stringent operational requirements on current desalination plants, and will require more plants to be built, which will in turn escalate the demand for energy and fuel.

Using renewable energy to power desalination processes is one of the sustainable solutions to provide potable water in water-scarce regions [7]. Solar energy is the most abundant renewable energy source and is highly suitable for powering both thermal and membrane processes. A number of studies have highlighted the importance and suitability of solar desalination for the Middle East and North Africa region (which is one of the most water-scarce regions globally) [8, 9, 10]. There is a need to assess the techno-economic feasibility of solar desalination plants in different countries so that informed decisions can be made.

The coupling of solar energy technologies, such as concentrated solar power and water desalination, is still in the research and development phases, and hence extensive research is needed in this field [11]. Numerous research studies investigated the challenges and opportunities for making solar energy powered desalination feasible [12, 13, 14, 15, 16, 17]. Among the currently developed solar desalination technologies, solar-thermal driven MED is possibly the most suitable for large-scale implementation owing to its superior thermodynamic and heat transfer performances (as compared to the MSF process), and lower levelised cost of water (LCOW) [18, 19].

## 2 Background

MED is a thermal desalination process wherein seawater is desalted by boiling at successive effects. The latent heat of an external heat source is used to boil a fraction of the feedwater in the first effect. This generates water vapor and brine at the saturation pressure. The brine is circulated to the next effect and partially boiled using the latent heat from the vapor generated in the preceding effect. This, subsequently, causes condensation of the water vapor and more distillate production. Each effect in the MED system is kept at a pressure and temperature lower than the preceding effect. This ensures that the brine will boil at a lower temperature. The utilization of the generated vapor in each effect that acts as a heating source for the next effect is an energy recovery method that distinguishes the MED and MSF processes. Thermal desalination processes are characterized using the gain output ratio (GOR) and the performance ratio (PR). The GOR is the mass flow rate ratio of the distillate to the heating steam as defined in Eq. (1). The GOR is always equal to, or less than, the number of effects. Using the GOR, however, does not provide any information about the enthalpy difference of the steam across the desalting point, the quality of the steam, or the pumping energy [20]. The performance ratio (PR) is a useful metric for these parameters. The PR is the amount of desalted water produced by condensing steam at an average temperature corresponding to a latent heat of 2330 kJ/kg.

(1) $$\text{GOR}=\frac{\text{mass}\;\text{flow}\;\text{rate}\;\text{of}\;\text{distillate}}{\text{mass}\;\text{flow}\;\text{rate}\;\text{of}\;\text{heating}\;\text{steam}\;}$$

(2) $$\text{PR}=\;\frac{\text{mass}\;\text{flow}\;\text{rate}\;\text{of}\;\text{distillate}\;}{Q_{d}/2330}$$

**Fig. 1:**
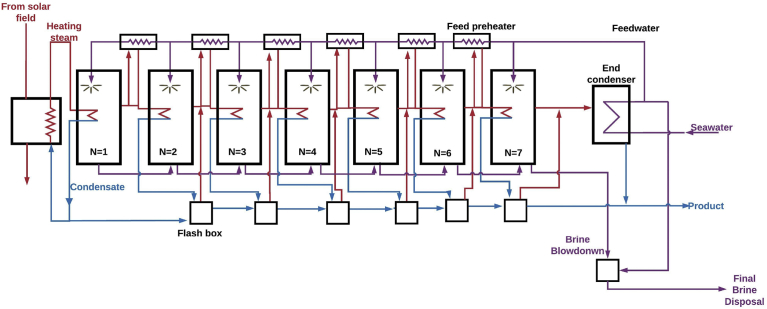
A seven-effect, parallel feed, horizontal stack MED unit. In this schematic, the MED unit is supplied with saturated steam from a solar collector.

In Eq. (2), Q_d_ is the heat supplied to the desalting unit in kW. Fig. 1 shows a seven-effect parallel feed horizontal stack MED unit that is supplied with heat from a solar collector. The MED process has a number of advantages over the MSF process [20]. MED can be carried out at a low top brine temperature (TBT), and hence, low-grade process steam can be used to power the process. A low TBT also minimizes the likelihood of scale formation. Furthermore, the MED system is more flexible than the MSF system. For example, an eight-effect MED plant with a GOR of 7.5 and a capacity of 1 million imperial gallons per day (MIGD) can be rearranged to work as a 4-effect plant with double the capacity (2 MIGD) but a lower GOR. Moreover, the MED process is more responsive to the enthalpy of the heating system than the MSF and can change its distillate capacity accordingly. The MED process also has a lower energy consumption rate than the MSF process due the utilization of the horizontal tube evaporator [21].

The coupling of solar energy and renewable energy with desalination systems is a new and an active area of research. This is because numerous barriers still exist before commercialization and mass-scale adoption. Currently, less than 1% of global water desalination capacity comes from renewable energy sources [22]. This is because renewable energy technologies are costlier than fossil fuels in terms of capital expenditure. However, current research suggests that the technical capacity of solar-thermal driven MED is more than 5000 m^3^/day [22, 23]. This indicates tremendous research and development opportunities.

There are a few studies that focused on the entire plant design for solar thermal MED systems. Among the important studies is the MED pilot plant at Plataforma Solar de Almeria (PSA) in Spain. This project was initiated in 1988 and included many experimental and modeling studies. The pilot plant used compound parabolic collectors (CPC), a 14-effect MED plant with vertical tubes and an absorption heat pump. Among the published results is that the use of a heat pump optimized the heat consumption of the system [24]. Sharaf et al. [15] carried out a thermo-economic analysis for two configurations of a solar thermal MED plant with and without a steam ejector, and considered the co-generation of desalted water (DW) and electric power (EP). The solar field consisted of parabolic trough collectors (PTC). This study assumed a fixed solar direct normal irradiance (DNI) input to the model. The study concluded that producing DW only is better than cogeneration in terms of LCOW and the solar field area. No extensive analysis of the solar field was provided in this study. A research work on the PSA pilot plant devised a control strategy for the temperature of the heat transfer fluid (HTF) leaving the solar field [25]. The optimization was carried out based on economic objectives. The authors found that by adjusting the outlet temperature of the solar field, plant running costs were reduced. De La Calle et al. [26] developed a nonlinear dynamic model using the Modelica package for the MED pilot plant at the PSA. The aim was to simulate the thermal performance of the first effect of the plant. Results have yielded good agreement between the model and the actual test results.

A few studies have considered the use of a linear Fresnel collector as an energy source for a MED plant. LFCs are inherently more suitable for MED than PTCs because they are low-temperature collectors and hence suit the low temperature steam requirements for MED which is around 70–78 °C. Linear Fresnel collectors are also more compact (ie. They have a smaller mass per unit area) than the PTC and are less costly. We consider that more research should be conducted with a focus on the use of the LFC with the MED process, and on the optimization of the solar field size. The solar field is effectively the energy source for the MED process, and hence requires significant development to suit the thermodynamic requirements of this process. The main thermodynamic requirement is the constant production of superheated or saturated steam at the design temperature and pressure of the TVC. Hamed et al. [27] carried out an experimental study for coupling LFCs with a MED-TVC system. Onsite measured solar radiation data was obtained from the city of Al-Jubail in Saudi Arabia. This study showed an improved optical efficiency for the tested LFC as compared to the Novatec and Industrial Solar LFC system. The study concluded that it is highly recommended to set the outlet temperature of the HTF to a low value so as to increase the thermal efficiency of the solar field. Furthermore, Askari and Ameri [28] also carried out a similar study in which the performance of a LFC solar field coupled to a MED plant was modeled using MATLAB and the System Advisor Model (SAM) software. This hybridized system incorporated a natural gas boiler. This study also investigated the effect of partial defocusing of the solar field on the solar share (fraction of the thermal energy to the MED delivered from the solar field). Results showed that the minimum LCOW occurs at the lowest solar share (27.54%). More about state of the research in solar-driven multi-effect distillation can be found in the review by Alhaj et al. [29].

Ensuring that the solar field's annual performance is unaffected by intermittent solar radiation is also a key challenge. Most research efforts have attempted to solve this problem using thermal energy storage (TES). However, these systems are very costly, and many researchers have reported that they result in increasing the specific water cost. There is a need for an in-depth economic analysis that uses data that accurately represent the actual costs. By doing so, we can determine which components of the solar desalination plant need to be optimized so as to reduce the LCOW. If we compare solar desalination with conventional desalination at present, the outcome will be in favor of the latter because of its lower capital costs and higher commercial maturity. Since solar desalination is a relatively new area of research, a lot of optimization and innovation will be needed before this technology can become competitive from the economic viewpoint. Table 1 summarizes a number of research components and modeling assumptions in solar-driven MED research, as found in the literature.

**Table 1:** Specific research components/modeling assumptions endorsed in solar-thermal driven MED.

Research Component	Reference
**Solar radiation assumption**	
Fixed value for solar radiation assumed in model	[15, 27, 30]
Hourly variable solar radiation data used in model	[28, 31, 32]
**Type of solar collector**	
Use of linear Fresnel collectors	[27, 28]
Use of parabolic trough collectors	[15, 31, 33, 34, 35, 36]
**Thermal energy storage (TES)**	
Use of thermal energy storage	[27, 28, 30, 31, 33, 35, 37, 38]
No thermal energy storage	[15, 32, 34, 36, 39, 40]

## 3 Hypothesis

The current work proposes an operational strategy for a solar field supplying thermal power to a MED-TVC pilot plant. The aim of this work is to a) simulate the performance of the solar field under Qatar's climatic conditions, and b) present a control strategy that reduces electric pumping power in the solar field. The first part of this paper is a brief introduction and an overview of solar-thermal driven MED, its current status, major research works, and a description of the objectives of this work. The second part outlines the research methodology and model development stages. The third part presents the results and discussion of our findings. Finally, the paper is concluded by highlighting key results and by outlining steps to be taken in the future.

## 4 Materials and methods

**Fig. 2:**
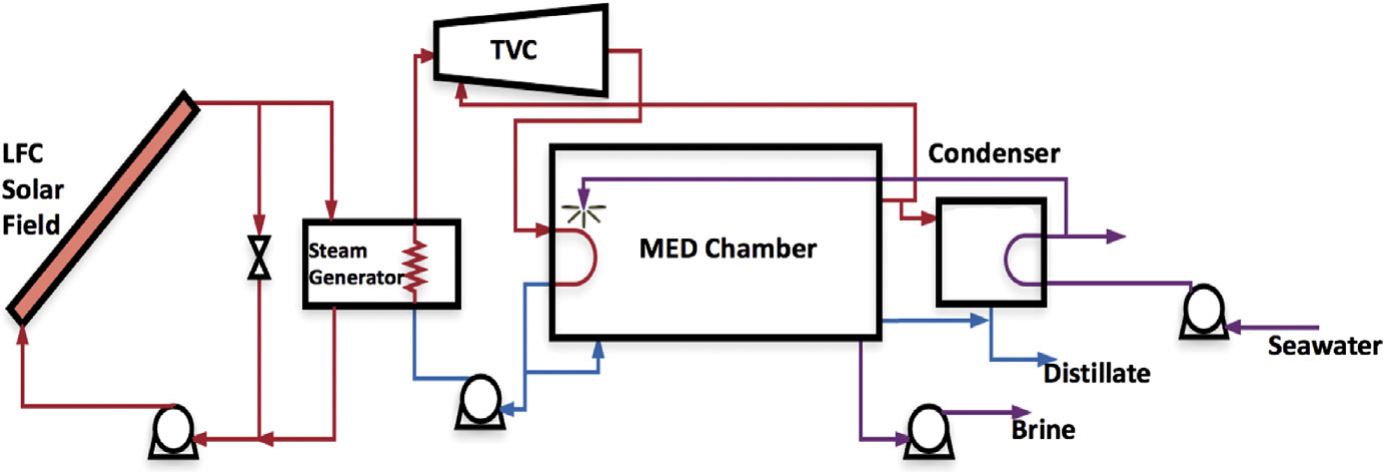
The proposed MED-TVC pilot plant driven by solar thermal energy.

Our plant is a MED-TVC system coupled to an LFC solar field that supplies thermal energy through an HTF loop. The HTF is pressurized water. Fig. 2 shows a schematic of the solar-thermal driven MED-TVC pilot plant. The plant is a seven-effect MED-TVC desalination plant using parallel feed flow, feed preheaters, flash boxes, and an end-condenser.

**Fig. 3:**
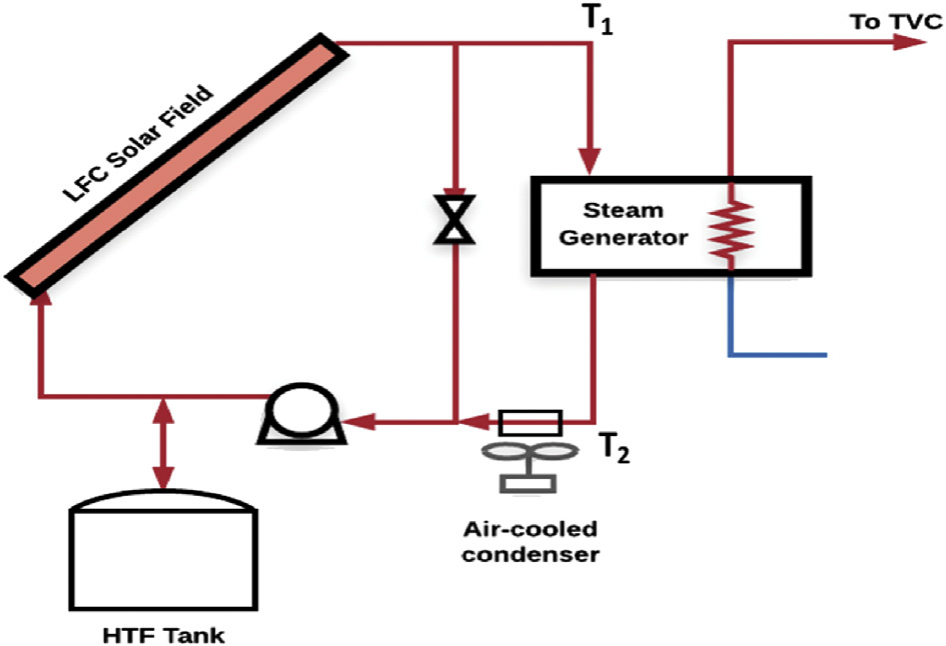
Schematic of the solar field. T_1_ and T_2_ are the HTF inflow and outflow temperatures respectively.

The solar field section has two loops, namely, a bypass loop, and a HTF loop. The bypass loop is used to recirculate a specific amount of mass flow of the HTF when the field power is more than the required thermal power at the steam generator. A variable speed pump controls the mass flow rate in the solar field section. The steam ejector (or TVC) uses motive steam generated at the steam generator that is supplied with thermal energy from the solar field. Saturated steam exiting the TVC will be used as a heating source to the first effect of the MED unit. The condensed heating steam is returned into the solar field's cycle's heat exchanger after leaving the first effect, except for the entrained vapor fraction that will be returned into the first flash box. The geometry of the solar field was based on the collector developed by Industrial Solar GmbH. The specifications of this system are listed in Table 2. Fig. 3 shows a schematic of the solar field module.

**Table 2:** Specifications of the linear Fresnel collector [41].

Parameter	Value
Number of modules	8
Aperture area of single module	22 m^2^
Field aperture	8 × 22 = 176 m^2^
Focal length	∼4.5 m
Primary mirrors	Toughened white glass mirrors
Mirror support structure	Steel, Aluminum
Absorber tube	SCHOTT PTR70
Total absorber length	64.96 m
Absorber diameter	3.47 cm
Secondary reflector	Highly reflective Aluminum
Weight	28 kg/m^2^
Maximum pressure	20 bar
Maximum temperature	200 °C
Maximum optical efficiency	66%

### 4.1 Model

The main assumptions used for modeling are:

• All components are operating at steady state conditions.
• Hourly solar radiation is constant.
• Convective thermal losses from the receiver are neglected. This is because the pressure in the receiver glass envelope is nearly a perfect vacuum; 0.001 mbar [42].
• There is a very small pressure drop in the piping of the LFC system because the receiver's length is small.
• Plant start-up and shut down is not considered in the analysis.
• Hourly collector optical efficiency is constant (0.66). This conforms with the manufacturer's specifications and was also validated by our calculation of the hourly optical efficiency (under Qatar's DNI for a North-South orientation) using SAM software.

The model inputs include the DNI data for Doha, Qatar, the hourly optical efficiency for the linear Fresnel collector, thermophysical properties of pressurized water at 6 bar (the HTF), and the design temperature difference at the steam generator: set at 10 °C (inflow temperature: T_1_ = 150 °C and outflow temperature: T_2_ = 140 °C). The DNI was measured using a Kipp and Zonen CHP1 pyroheliometer with an uncertainty of 2%. The thermophysical properties of water (density and specific heat capacity) were found using the libraries in the Engineering Equation Solver (EES) software. The model's outputs are the hourly collector field power, the hourly mass flow rates of the HTF at different solar intensities, the solar field electric energy consumption, and the distillate produced from the desalination plant.

The plant's equations relating to the solar field section (shown in the governing equations section below) were implemented in the EES software. These equations are solved simultaneously in EES using Newton's method with a convergence residual of 10^−6^. The equations describing the MED-TVC system were based on another EES model developed and validated by the authors [43]. This model comprises: the energy, mass, and salt balance equations for a parallel-feed MED plant with brine recirculation, preheaters, and flash boxes. The relations by Hassan and Darwish were used to model the TVC [44]. A total of 339 equations are used in the model.

The equations describing the model include the solar insolation and the solar field performance. Ground measurements of DNI in Doha, Qatar for the year 2016 were used to run the model. The solar field's governing equations were based on an energy balance.

A major aim of our work is to develop a model that simulates the realistic performance of the solar field, thereby imposing the need to use real and not satellite-derived solar radiation data which may have large errors. We used ground measurements of DNI in Doha in 2016 to derive a function approximation for the hourly DNI for every month of the year at Doha, Qatar (25.17° N, 51.32° E). The original DNI values were in 1-minute intervals for the entire year. We sampled the dataset into hourly values and then plotted them to derive a function approximation using curve-fitting in EES. The derived function approximations for DNI are unique to Qatar and can be used for assessing the hourly performance of various solar collectors. These function approximations are given in [43].

The optical efficiency is an important metric in assessing the performance of concentrating solar collectors. The optical efficiency is a measure of the efficiency of the mirrors in reflecting the incident solar radiation at the receiver with minimal deviations. It is directly affected by the cleanliness of the mirrors, tracking errors of the mirror drive, cosine of the incident angle, mirror and receiver shading, soiling factor, intercept factor, and specular reflectance of the mirror material. The general mathematical form for the optical efficiency is given in Eq. (3):

(3) $${\text{η}}_{\text{optical}}=\;\frac{\text{Thermal}\;\text{energy}\;\text{absorbed}\;\text{by}\;\text{the}\;\text{receiver}}{\text{Incident}\;\text{solar}\;\text{energy}}$$

We computed the hourly optical efficiency using SAM software based on the incidence angle modifier (IAM) method. The IAM is a correction factor method that accounts for the effects of the transversal (Φ_T_) and longitudinal (Φ_L_) incidence angles on the optical efficiency. The optical efficiency is related to the IAM by Eq. (4):

(4) $${\text{η}}_{\text{optical}}={\;\text{η}}_{\text{max}}\;\times {\;\text{IAM}}_{\text{T}}\;\times {\;\text{IAM}}_{\text{L}}$$

where η_max_ is the maximum optical efficiency at 0° incidence (provided by the manufacturer as 0.66), and IAM_T_ and IAM_L_ are the transversal and longitudinal IAMs, respectively.

The solar field performance is expressed by the power delivered by the HTF (we call this term Q_field_). In addition, we are interested in calculating the HTF mass flow rate at different solar intensities and investigating the possibility of reducing HTF mass flow rate so as to reduce the electrical pumping energy. The receiver incorporated in the LFC setup at the STF is an evacuated tube (SCHOTT PTR70) which has a diameter of 70 mm and length of 64.96 m. The generic relation for the useful thermal energy absorbed by the HTF in the solar field is defined in Eq. (5):

(5) $$Q_{u}=m_{htf}\;\times \;c_{p}\;\times \;{\text{Δ}}_{HTF}$$

where m_htf_ is the mass flow rate of the HTF in kg/s, c_p_ is the specific heat capacity in kJ/kgK, and ΔT_HTF_ is the temperature difference between the inlet and outlet streams of the HTF. The thermal power delivered by the solar field in kW is given by:

(6) $$Q_{\text{field}}=\;\frac{\text{DNI}\;\times {\;\text{A}}_{\text{field}}\;\times {\;\text{η}}_{\text{optical}}\;–{\;\text{Q}}_{\text{loss}}}{1000}$$

DNI is the direct normal irradiance (in W/m^2^), A_field_ is the aperture area of the LFC (in m^2^), and Q_loss_ is the total thermal loss from the receiver (in W). In Eq. (6), DNI and η_optical_ are evaluated on an hourly basis. Q_loss_ is a function of the receiver's average temperature, which is given by Eq. (7):

(7) $$T_{\text{receiver}}=\frac{T_{1}+T_{2}}{2}+10$$

T_1_ and T_2_ are the HTF inflow and outflow temperatures respectively (refer to Fig. 3). We assumed that the receiver's surface temperature is a few degrees above the average of T_1_ and T_2_. We assumed that the receiver's temperature is 10 °C above the average HTF's temperature as validated by the National Renewable Energy Laboratory (NREL) [45]. Given the value of T_receiver_, Q_loss_ can be estimated based on [42]:

(8) $$Q_{\text{loss}}=8.56993\;\times \;exp^{\left(0.00844872\times T_{\text{receiver}}\right)}\;\times \;L_{\text{receiver}}$$

where L_receiver_ is the length (m) of the Schott PTR70 receiver. In Eq. (8), the Q_loss_ term is effectively the radiative heat loss which is a function of the absolute temperature of the receiver. In our model the convective heat loss was neglected because the space between the receiver and its glass envelope is almost a perfect vacuum (0.001 mbar [42]). We can infer from Eqs. (7) and (8) that operating the LFC system at a high temperature differential will result in more thermal losses and hence a lower thermal efficiency. This would happen in large solar fields where the HTF is recirculated resulting in a temperature rise of up to 200 °C. The experimental work by Hamed et al. on a LFC system tested under the climate conditions of Saudi Arabia demonstrates the above conclusion [27]. In the aforementioned study, the authors found that setting the outflow temperature of the HTF to a low value results in a higher thermal efficiency. The influence of this concept on our model is that the as the thermal efficiency of the LFC system reduces, less heat is transferred to the MED evaporator and hence the distillate production reduces. The design mass flow rate of the HTF in the steam generator was estimated based on Eq. (9). The design required thermal power (Q_req_) is 40 kW_th_ and m_design_ is equal to 0.93 kg/s.

(9) $${\text{m}}_{\text{design}}=\;\frac{{\text{Q}}_{\text{req}}}{{\text{C}}_{\text{p}}\times \;\text{Δ}T_{steam\;generator}}$$

ΔT_steam generator_ is 10 °C. The proposed operational strategy is to preheat the HTF by recirculation in the bypass loop until T_1_ reaches 150 °C. The HTF will then be allowed to recirculate in the steam generator's loop at a constant mass flow rate of 0.93 kg/s. This will ensure constant thermal power delivered to the MED-TVC (through the steam generator), as long as the field power (Q_field_) is greater than 40 kW. If the field power is less than 40 kW, the plant will not be operational. On the other hand, if the field power is more than 40 kW, the excess power will be diverted through the bypass loop. The HTF tank in Fig. 3 is used to the control the amount of the HTF within the plant loops. Further, an air-cooled condenser is integrated in the cycle to ensure the HTF inflow temperature is regulated. The specific mass flow rate in the bypass loop (referred to as m_bypass_) is computed on an hourly basis using Eq. (10):

(10) $${\text{m}}_{\text{bypass}}=\;\frac{{\text{Q}}_{\text{field}}\;-{\;\text{Q}}_{\text{req}}}{{\text{c}}_{\text{p}}\times \;\text{Δ}T_{steam\;generator}}$$

The total mass flow rate in the pump is given by Eq. (11):

(11) $$m_{\text{pump}}=m_{\text{design}\;}+\;m_{\text{bypass}}$$

The pumping power is related to the mass flow rate in accordance with Eq. (12):

(12) $${\text{W}}_{\text{pump}}={\text{m}}_{\text{pump}}\times \;\frac{\text{Δ}{\text{P}}_{\text{htf}}\;}{{\text{η}}_{\text{pump}}\times {\text{ρ}}_{\text{htf}}}$$

where m_pump_ is the pump mass flow rate in kg/s, ΔP_htf_ is the pressure drop of the HTF (assumed as 0.3 bar), ρ_HTF_ is the density of the HTF in kg/m^3^, and η_pump_ is the pump efficiency, which was assumed to be equal to 0.9. If we consider the solar-driven MED-TVC plant as one unit, then an increase in the pumping power (W_pump_) in the solar field effectively corresponds to a higher specific power consumption (SPC) for the entire system. SPC is a common metric used to assess desalination systems and is calculated using Eq. (13):

(13) $$\text{SPC}\;=\frac{{\text{W}}_{\text{electric}}}{\text{Distillate}}$$

where W_electric_ is the pumping power in the entire system (solar field and desalination plant) in kW. A high specific power consumption would result in higher operational costs. Further, if this energy comes from fossil fuels, then this implies a higher environmental footprint for the solar-desalination plant. It is important, hence, to reduce the specific power consumption as much as possible.

## 5 Results & discussion

The overall system specifications and EES model results are shown in Table 3. This table reflects the performance of a small scale solar-driven MED-TVC plant located in Qatar. Based on the solar field's specifications and the capacity of the desalination plant, the specific yield is 8.5 m^3^ of freshwater per m^2^ of the solar collector per year in Qatar. Further, this plant would operate for 2769 hours per year. The validation of the MED-TVC system results and the detailed comparisons with similar systems can be found in our paper [43].

**Table 3:** Specifications and performance of the simulated solar-driven MED-TVC plant.

Parameter	Value
**Solar Field**	
Aperture area (m^2^)	176
HTF	Pressurized water
Receiver length (m)	64.96
HTF temperature increase (C)	10
**MED-TVC chamber**	
Number of effects	7
Top brine temperature (°C)	65
Feed type	Parallel flow
^i^Seawater temperature (°C)	30
^i^Seawater salinity (g/kg)	48.2
^i^Design ΔT in end condenser (°C)	7
^i^Brine temperature in the last effect (°C)	40
Motive steam pressure (bar)	3
Compression ratio	4.4
Mixing ratio	3.0
**Performance Metrics**	
GOR	8.3
Specific thermal power (kWh/m^3^)	81.3
Specific power consumption (kWh/m^3^)	2.1
Specific area (m^2^/kg/s)	282
Productivity (kg/h)	577.1
Recovery Ratio	0.29
Brine disposal temperature (°C)	38
Brine disposal salinity (g/kg)	52.7
Summer daily operating hours (h)	8
Winter daily operating hours (h)	6

**Fig. 4:**
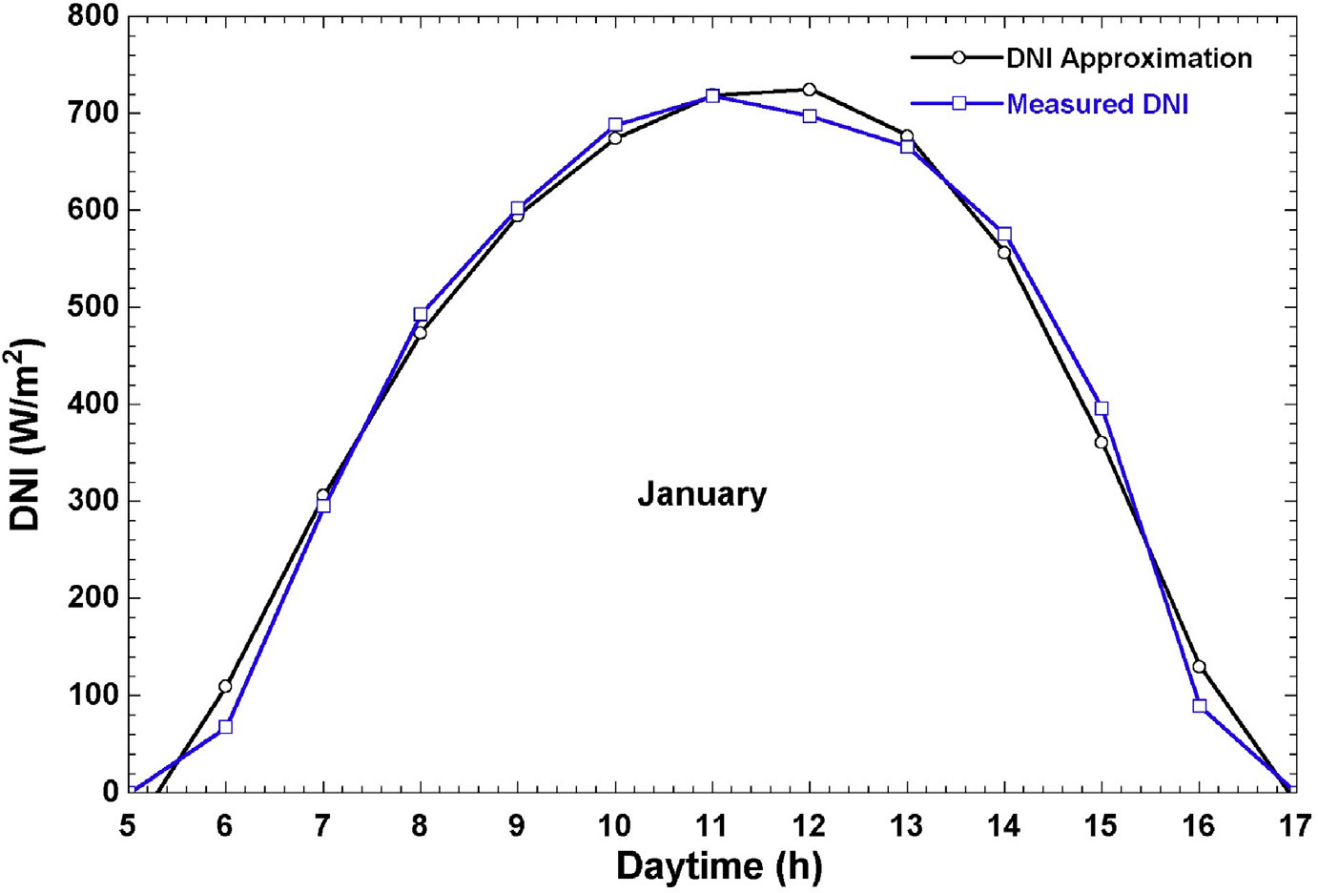
Comparison of measured solar direct normal irradiance and the function approximation modelled in the Engineering Equation Solver.

Fig. 4 shows the real DNI data and the function approximation for a typical day in the month of January. This figure shows that the DNI is a bell-shaped profile with a maximum intensity of approximately 700 W/m^2^, which occurs between 11 am–12 pm. Fig. 4 shows that the polynomial function approximation closely resembles actual ground measurements and hence it valid to use.

**Fig. 5:**
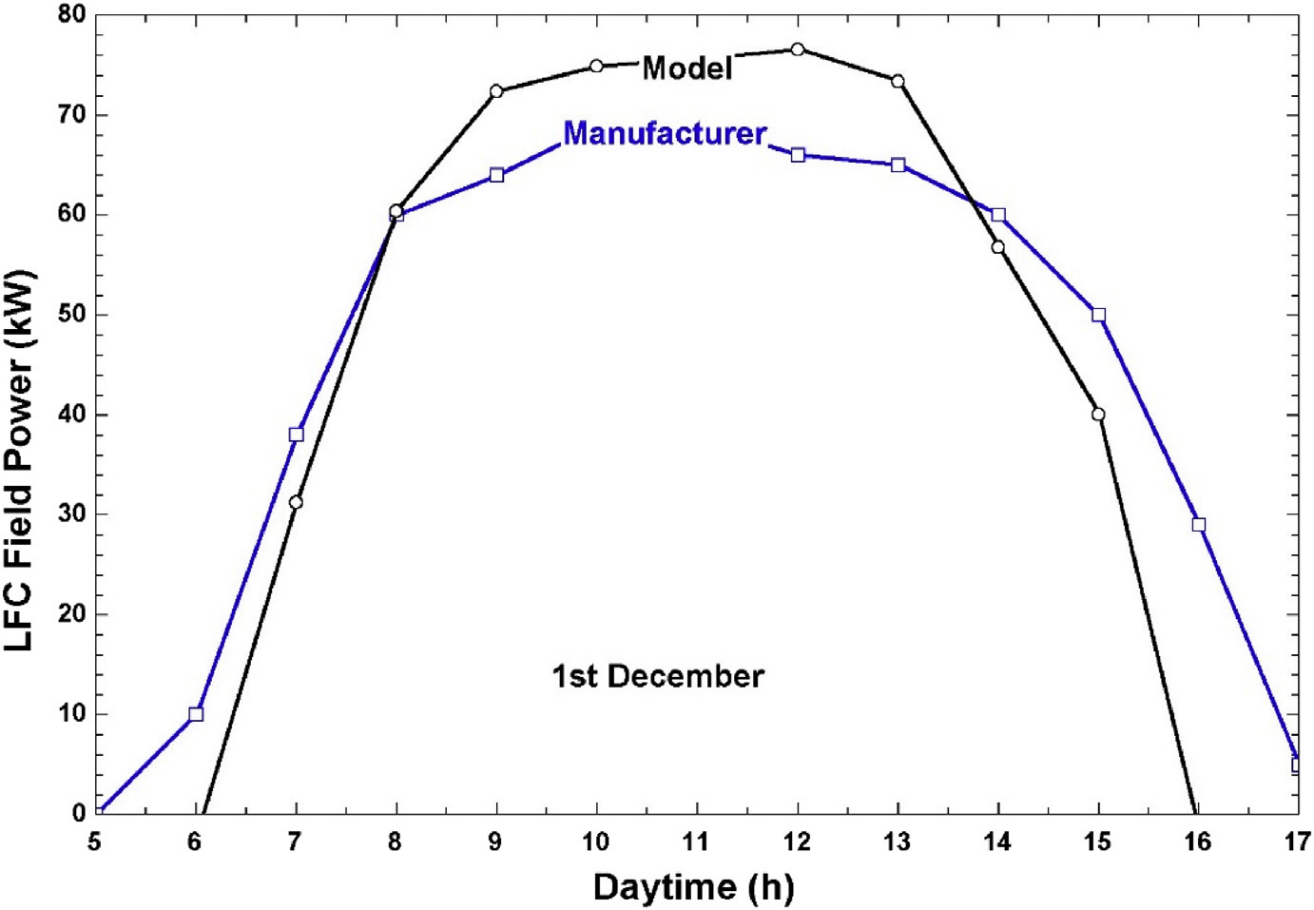
Solar field's hourly thermal power in 1st December.

Using Eq. (6), we computed the hourly solar field power, which will be the input power at the steam generator. Fig. 5 show the hourly field power from our model and the comparison with the manufacturer's results for the same system size for the month of December under the climate conditions of Doha, Qatar. This figure shows a close match between the model and the manufacturer's results. The deviation between the model and the manufacturer's results for three months in the year is shown in Table 4. Table 4 shows a maximum absolute error of 8% which is acceptable. These results show that the model we developed is valid and predicts to a reasonable degree of accuracy a real solar field's performance.

**Table 4:** Model validation results.

Month	Model's results - Average field power (kW)	Manufacturer's results - Average field power (kW)	Absolute error (%)
March	42.9	45.3	5.3
April	47.1	51.2	8
December	42.6	45	5.3

**Fig. 6:**
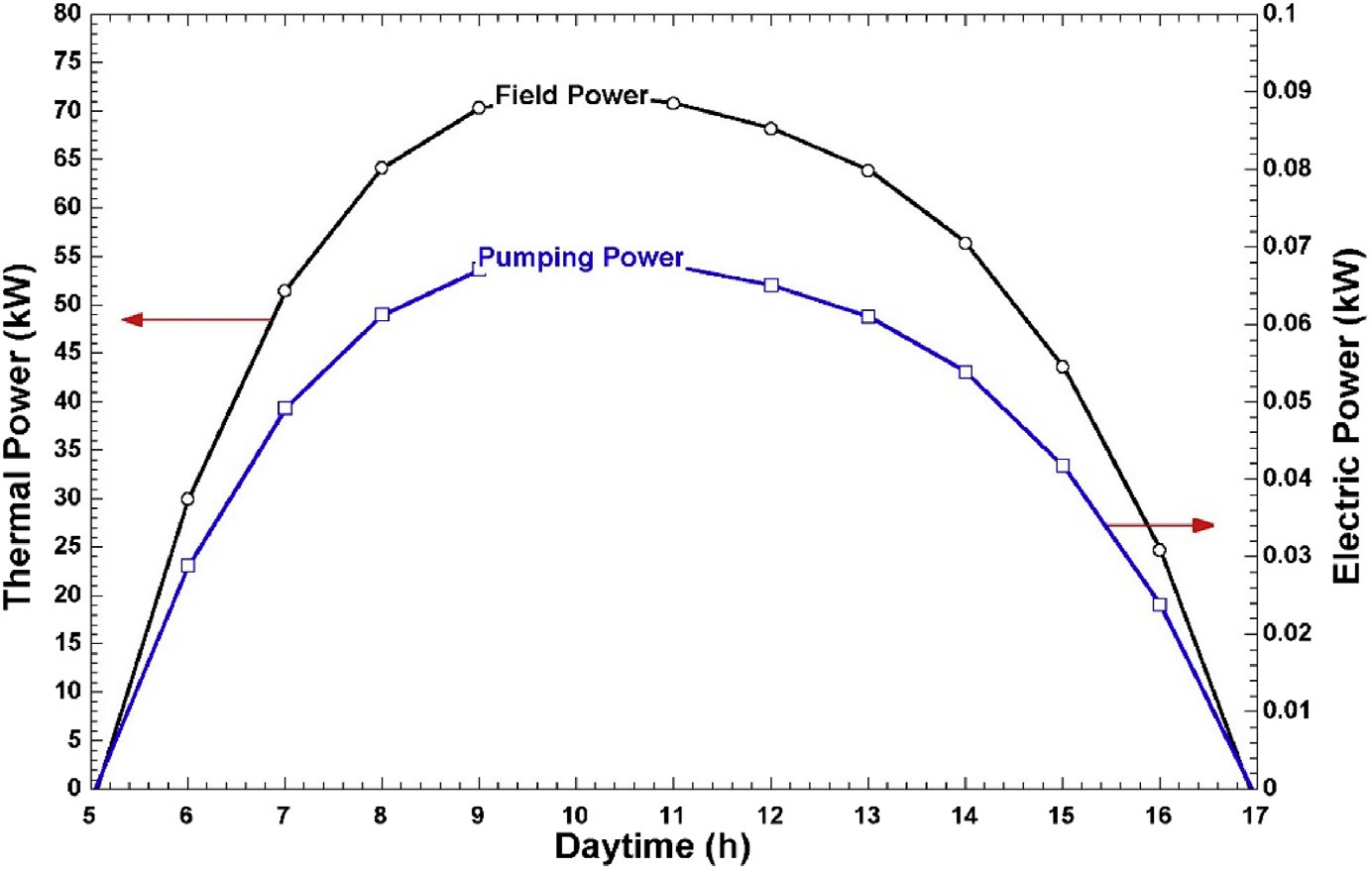
LFC field power and pumping power in April.

**Fig. 7:**
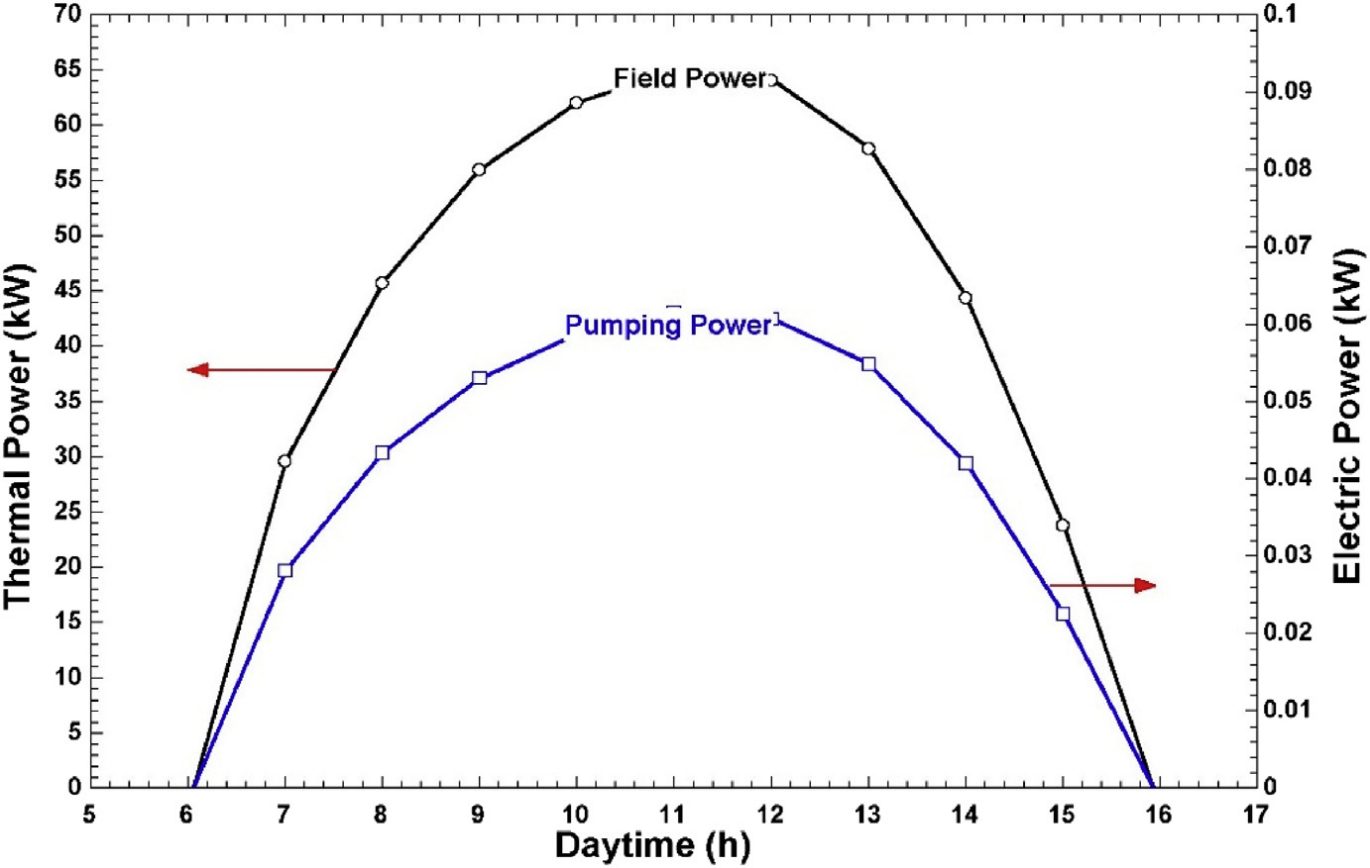
LFC field power and pumping power in December.

Thermal desalination systems are designed to operate at constant power. However, hourly solar intensity is variable. To overcome this problem and ensure constant power is delivered to the steam generator, the HTF mass flow has to be regulated. Fig. 6 shows the field power and the corresponding pumping power during the operational period of the solar field in summer (April). As shown in Fig. 6, when the field power increases, the HTF mass flow also increases, which results in higher electrical energy consumption. The daily electrical energy consumption in summer is 0.49 kWh. The maximum mass flow of HTF in this system is 1.684 kg/s and it occurs at 10.00 am. Fig. 7 shows the solar field's winter (December) profile for thermal power and electric pumping power. The daily electrical energy consumption in winter is 0.37 kWh while the maximum HTF mass flow rate is 1.51 kg/s occurring at 11 am.

### 5.1 Study area

The pumping power values in Figs. 6 and 7 may be small, but this is because of the scale of the LFC system we used. To overcome the problem of having to increase the mass flow rate as DNI increases, we employed the concept of collector defocusing. This implies the defocusing of n-collector rows, which will reduce A_field_, and hence Q_field_. We attempted to propose a recommended hourly focus ratio that reduces m_pump_ to the least possible value. We also investigated the effect of this control strategy on reducing pumping power. The focus ratio is given by Eq. (14):

(14) $$\text{Focus}\;\text{Ratio}\;\left({\text{r}}_{\text{foc}}\right)=\;\frac{\text{number}\;\text{of}\;\text{collector}\;\text{rows}\;\text{focused}}{\text{total}\;\text{number}\;\text{of}\;\text{collectors}}$$

When we incorporate r_foc_ in our model, the solar field's power (Q_field_) is now calculated using Eq. (15):

(15) $$Q_{\text{field}}=\frac{DNI\;\times \;A_{\text{field}}\;\times \;{\text{η}}_{\text{optical}}\;\times \;r_{\text{foc}}-Q_{\text{loss}}}{1000}$$

**Fig. 8:**
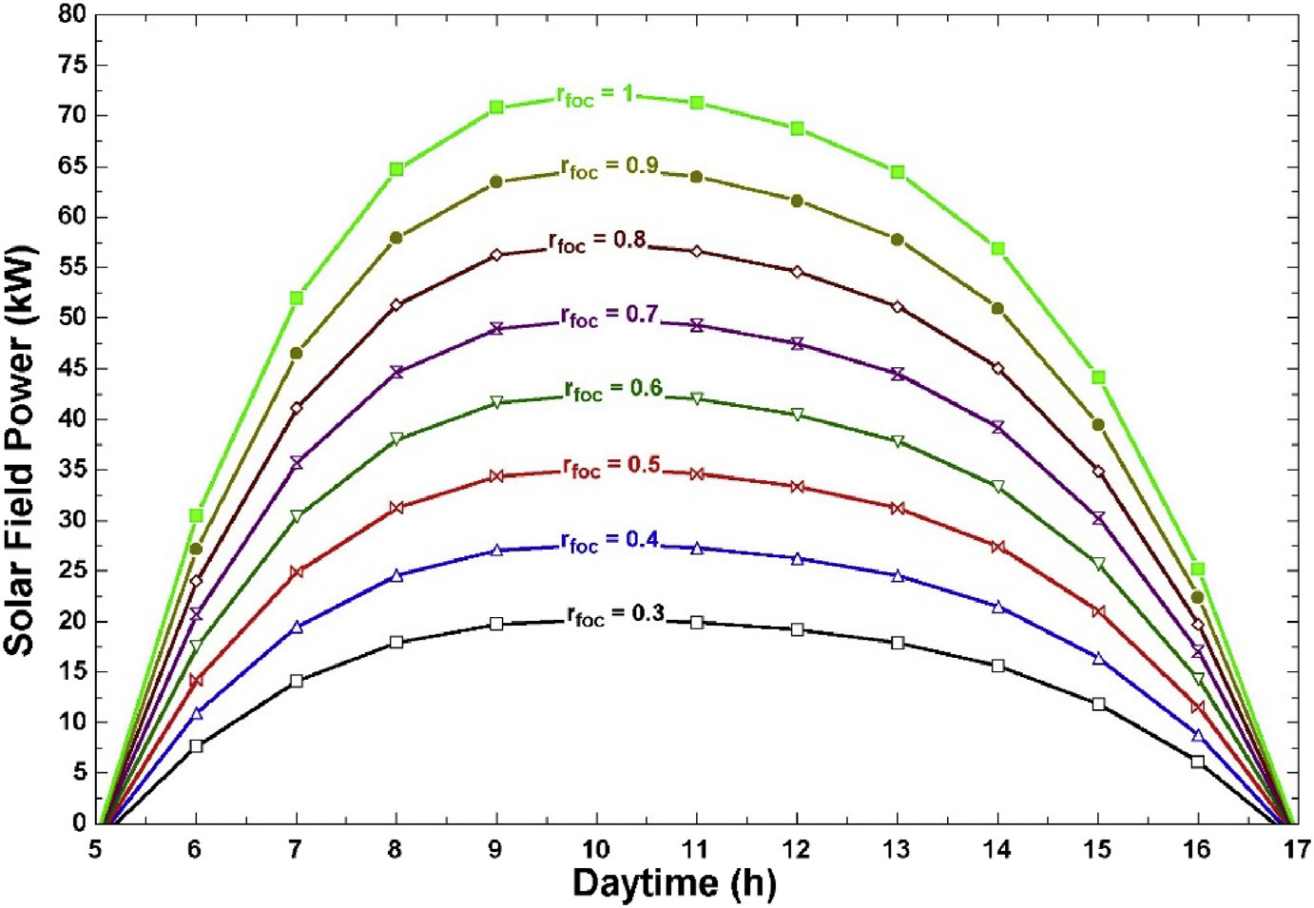
Hourly LFC field power at different focus ratios.

The total number of collector rows in our LFC setup is 10 and each collector can be controlled separately. The focus ratio thus varied from 1 to 1/10, with the value of 1 indicating the state where all the collectors were focused towards the receiver, and the value of 1/10 indicating that only one row was focused. Any change in the focus ratio will mean an equivalent change in the effective aperture area. A parametric study was carried out to investigate the effect of different focus ratios on the LFC field power, total HTF mass flow rate, and pumping power. Fig. 8 shows the hourly solar field power for different focus ratios. As this figure shows, the acceptable focus ratios are: 0.7,0.8,0.9, and 1. Any lower focus ratios will result in a field power less than the required amount by the desalination system (40 kW).

**Fig. 9:**
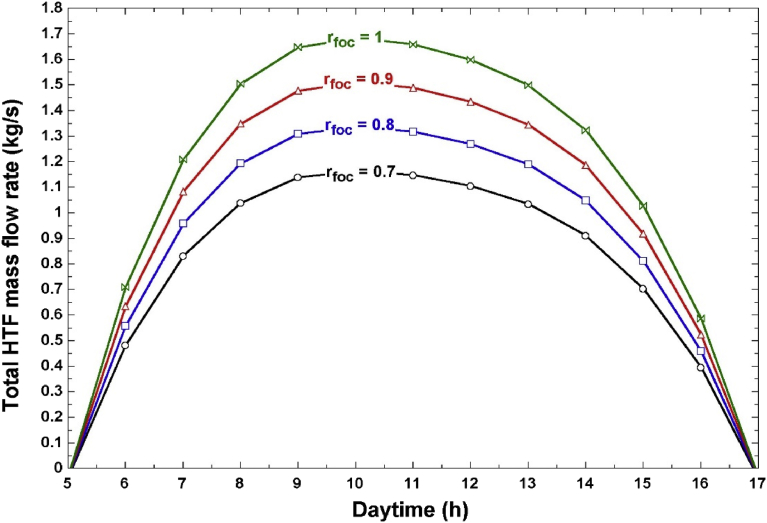
HTF mass flow rate at different focus ratios.

Fig. 9 shows the total HTF mass flow rate (m_pump_) for the acceptable focus ratios found from Fig. 8. This figure shows that using a focus ratio of 7/10 will keep the mass flow rate minimal. However, a focus ratio of 7/10 for the entire day will reduce the operating hours of the LFC field to approximately 6 hours as indicated from Fig. 8. Hence, there is a need to select the optimal hourly focus ratios. This will result in reduced pumping power without reducing the operational period.

By examining the hourly values of m_pump_ for each focus ratio in Fig. 9, we selected the optimum hourly focus ratios and estimated the new pumping power for two representative months in the year (April and December). These values are shown in Tables 5 and 6. Based on the results in these tables, we infer that during the morning operation, the focus ratio must be high to allow a maximum concentration of the DNI to the receiver. As the DNI increases, the amount of excess thermal power increases, and hence we need to defocus more collectors. This results in a decreased focus ratio. At approximately 2–3 pm, the DNI reduces (which also reduces the total solar field power), and the focus ratio needs to be increased again to concentrate as much thermal energy as possible. Table 5 shows that during summer, an operating period of 9 hours is possible whereas in winter (December), only 7 hours of daily operation are possible due to the lower solar intensity.

**Table 5:** Recommended hourly focus ratios for the month of April.

Daytime (h)	DNI (W/m^2^)	Focus ratio	Pump mass flow rate (kg/s)	Pumping power (kW)
7	465.1	0.8	0.9573	0.039
8	574.6	0.7	1.039	0.042
9	627.8	0.7	1.139	0.046
10	640.9	0.7	1.164	0.047
11	631.6	0.7	1.147	0.047
12	609.6	0.7	1.105	0.045
13	572.4	0.7	1.035	0.042
14	507.3	0.8	1.049	0.043
15	397.6	1	1.026	0.042

**Table 6:** Recommended hourly focus ratios for the month of December.

Daytime (h)	DNI (W/m^2^)	Focus ratio	Pump mass flow rate (kg/s)	Pumping power (kW)
8	411.9	0.9	0.954	0.039
9	500.5	0.8	1.034	0.042
10	552.7	0.7	0.997	0.041
11	577	0.7	1.043	0.042
12	570	0.7	1.03	0.042
13	516.6	0.8	1.069	0.043
14	400.1	1	1.033	0.042

**Fig. 10:**
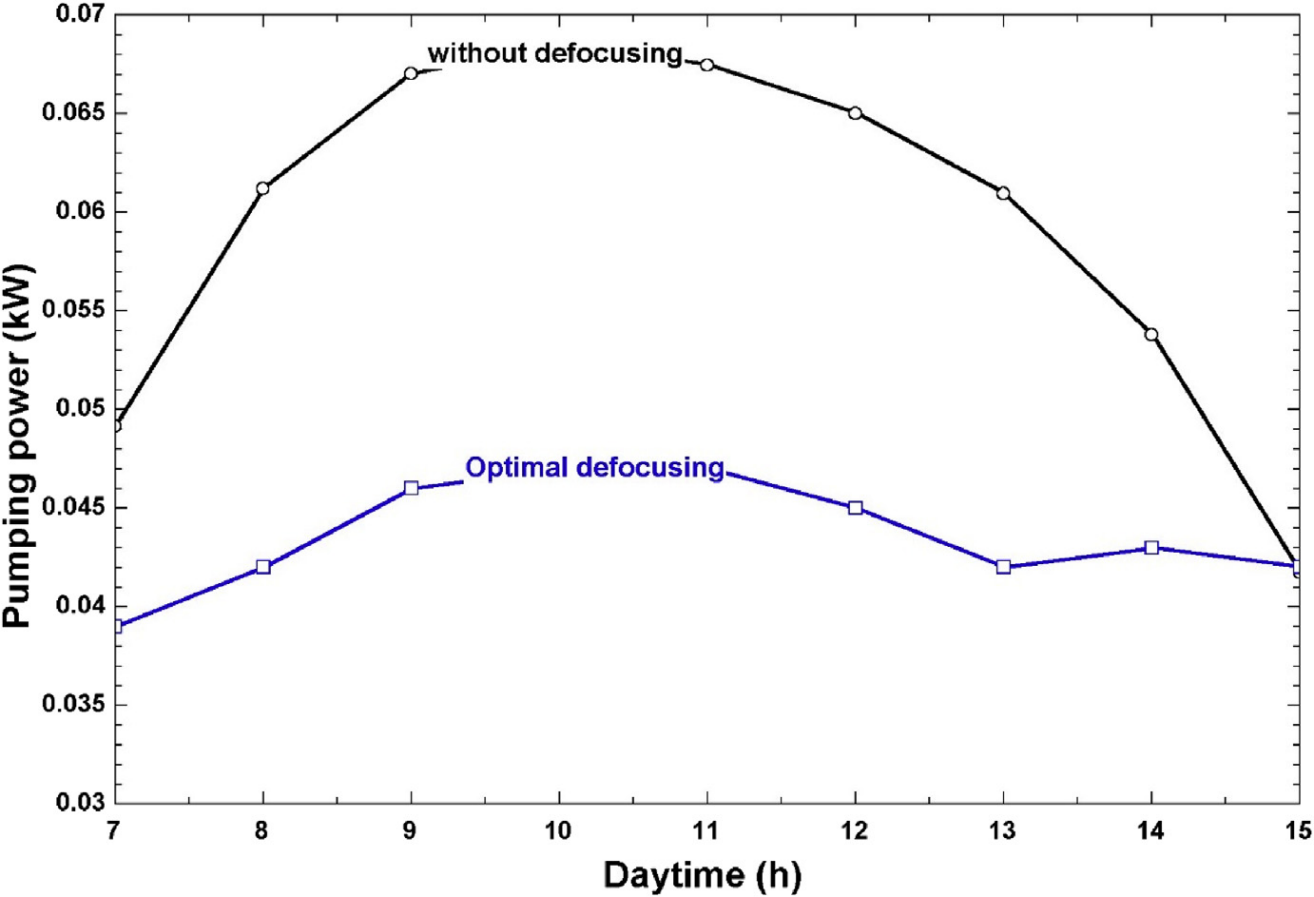
Hourly pumping power with and without defocusing (April).

**Fig. 11:**
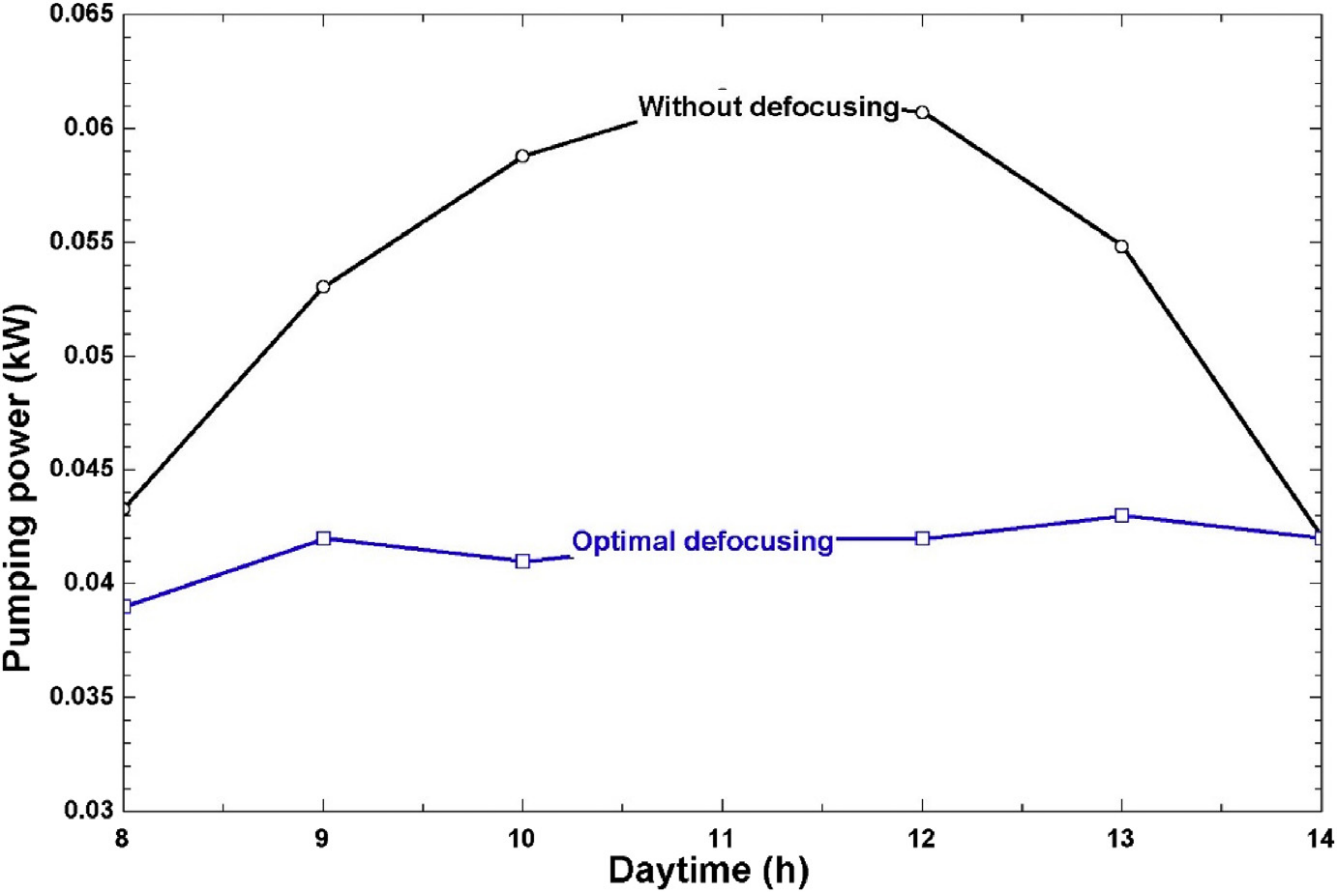
Hourly pumping power with and without defocusing (December).

The comparison between electric pumping power with and without defocusing is shown in Figs. 10 and 11. When no defocusing was applied on the solar field, the daily pumping energy in April was 0.59 kWh as shown in Fig. 10. However, when optimal defocusing was applied, this value was reduced to 0.35 kWh, which is equivalent to a 40% reduction in the daily pumping energy. This is an important result elicited from our work, and it highlights the considerable reduction in pumping power (and hence, the overall plant SPC) that can be achieved by hourly control of the solar collector rows. The same trend was observed for December where the daily pumping energy was reduced by 39% after optimal defocusing. The reduction in electrical energy consumption will also be reflected in lower energy costs and lower environmental impact for such a plant that partially relies on grid electricity.

## 6 Conclusions

Solar-driven desalination plants are potential alternatives to conventional fossil-fuel powered desalination plants. This work presented the simulation and performance of a LFC solar field driving a MED-TVC plant. The solar field's performance was evaluated using hourly optical efficiency and onsite measurements of DNI. Results from the solar field modeling showed that high DNI levels during summer allowed the solar field to be operational for 8 hours. The specific annual yield of the plant is 8.5 m^3^ of fresh water per m^2^ of solar collector under Qatar's climate. Using optimal defocusing of the solar field's collector rows, we were able to reduce the pumping energy by 40%. Our results highlight the versatility of LFC, as compared to non-concentrating collectors when used for thermal desalination. This work could be developed by model validation through experimental operation of a linear Fresnel collector. There is also a need to develop and test controllers for the solar field that minimize electrical energy usage and ensure stable plant operation by considering multiple parameters, such as the required outlet temperature, piping pressure drops, and fluctuations in the solar radiation due to cloud cover. The influence of optimal collector defocusing on the LCOW and the environmental impacts of solar desalination should be investigated further.

## Declarations

### Author contribution statement

Mohamed Alhaj, Sami G. Al-Ghamdi: Conceived and designed the experiments; Performed the experiments; Analyzed and interpreted the data; Contributed reagents, materials, analysis tools or data; Wrote the paper.

### Funding statement

This work was supported by a scholarship (210003978) from the Hamad Bin Khalifa University (HBKU), a member of the Qatar Foundation (QF). Any opinions, findings, and conclusions or recommendations expressed in this material are those of the author(s) and do not necessarily reflect the views of the HBKU or QF. The publication of this article was funded by the Qatar National Library.

### Competing interest statement

The authors declare no conflict of interest.

### Additional information

No additional information is available for this paper.
